# Serological Status of Vaccine and Hepatitis B Virus Exposure Among Children Under 5 and Aged 15–17 Years in Kampala, Uganda

**DOI:** 10.3390/livers4040039

**Published:** 2024-10-24

**Authors:** Fahad Muwanda, Edward Kiyonga, Joan Nambafu, Agnes Turyamubona, Hussein Mukasa Kafeero, Edgar Kigozi, Harriet Mupere Babikako, Enock Wekiya, Gerald Mboowa, David Patrick Kateete, Hakim Sendagire, Paul J. Norman, Bernard Ssentalo Bagaya

**Affiliations:** 1 Department of Immunology and Molecular Biology, School of Biomedical Sciences, Makerere University College of Health Sciences, Kampala P.O. Box 7072, Uganda; 2 Department of Medical Microbiology, Habib Medical School, Islamic University in Uganda, Kampala P.O. Box 7689, Uganda; 3 School of Medical Laboratory Technology, Kibuli Muslim Hospital Health Training Schools, Kampala P.O. Box 24548, Uganda; 4 Child and Family Foundation Uganda (CFU) Medical Center, Plot 816/7, Block 206 Mpererwe-Ttula Kawempe Road, Kampala P.O. Box 73243, Uganda; 5 Child Health and Development Center, School of Medicine, College of Health Sciences, Makerere University, Kampala P.O. Box 7072, Uganda; 6 The African Center of Excellence in Bioinformatics and Data-Intensive Sciences, Infectious Diseases Institute, College of Health Sciences, Makerere University, Kampala P.O. Box 22418, Uganda; 7 Integrated Biorepository of H3Africa-Uganda (IBRH3AU), Department of Immunology and Molecular Biology, School of Biomedical Sciences, Makerere University College of Health Sciences, Kampala P.O. Box 7072, Uganda; 8 Department of Medical Microbiology, School of Biomedical Sciences, Makerere University College of Health Sciences, Kampala P.O. Box 7072, Uganda; 9 Department of Biomedical Informatics, University of Colorado School of Medicine, 13001 East 17th Place, Aurora, CO 80045, USA; 10 Department for Research, BMK Medical Laboratory Services, Mityana P.O. Box 36639, Uganda; 11 Sub-Saharan African Network for TB/HIV Research Excellence (SANTHE), Nelson R. Mandela School of Medicine, University of KwaZulu-Natal, K-RITH Tower Building, Level 3, Durban 4001, South Africa

**Keywords:** hepatitis B virus, serological status, combo test, hepatitis B vaccine, Kampala-Uganda

## Abstract

**Background:**

Pediatric hepatitis B virus (HBV) serostatus remains variably characterized, hardly determined at times, or documented as part of national monitoring of the Extended Programs for Immunization (EPI).

**Methods:**

We cross-sectionally characterized the seroprevalence of HBV vaccine and/or infection status among 501 and 288 children <5 and 15–17 years old, respectively, in Kawempe Division, Kampala, Uganda, between May and August 2023. These children received HBV vaccination under the Uganda National Extended Program on Immunizations (UNEPI). Samples were qualitatively screened for hepatitis B surface antigen (HBsAg), hepatitis B surface antibody (HBsAb or anti-HBs), hepatitis B e antigen (HBeAg), hepatitis B e antibody (HBeAb or anti-HBe), and for hepatitis B core antibody (HBcAb or anti-HBc) using three different HBV Combo test rapid immunochromatographic diagnostic tests: Nova, Fastep, and Beright.

**Results:**

The seroprevalence of HBsAg, anti-HBs, HBeAg, anti-HBe, and anti-HBc was 1.52%, 27.75%, 0.88%, 0.63%, and 0.76%, respectively, for the combined study age groups. The HBsAg seroprevalence of 2.78% was almost 3.5-fold higher among adolescents when compared to the 0.8% observed in the under-5-year-olds. The qualitative seroprevalence of anti-HBs was 33.1% and 18.4% in the under-5 and among the 15–17-year-old study groups, respectively.

**Conclusions:**

The proportion of qualitatively detectable anti-HBs in both groups of vaccinated children is low and probably indicates reduced seroprotection. Consequently, a large proportion of children who received the hepatitis B vaccine under UNEPI may be at risk of HBV infection, especially adolescents. A booster dose of the Hepatitis B Vaccine may be required for adolescents.

## 1. Introduction

Chronic hepatitis B virus (HBV) infection continues to be a global public health threat. It is estimated that 296 million people are currently living with the virus [1]. The global prevalence of HBV infection is 3.9%, and the WHO Africa region remains substantially burdened at a prevalence of 3.0% [2,3]. In highly endemic countries such as Uganda, the national prevalence of HBV infection is 10% [4].

Many African countries integrated HBV vaccination into their national extended programs for Immunization (EPI) by 2002. In Uganda, such as most of Sub-Saharan Africa, the HBV vaccination schedule is at birth, 6, 10, and 14 weeks [5–8]. Immunization is conducted at all levels of public healthcare, at all private hospitals, and in accredited health facilities of private practitioners. However, following vaccination, pediatric HBV serostatus remains variably characterized or documented as part of the national EPI monitoring. Approximately 20% of immunocompetent HBV vaccine recipients fail to respond to the complete vaccination [9–12]. In addition, effective control measures for HBV infection and disease are underutilized. For example, HBV infection is generally underdiagnosed, and coverage of fully vaccinated children remains low, especially in low-income and high-burdened countries [1].

As is with other vaccines, the protective efficacy of the Hepatitis B vaccine is probably lower in the East African region when compared with the more developed countries. A recent study in Ethiopia has reported close to 5% seroprevalence of breakthrough HBV infection among fully vaccinated children of 5–12 years of age [13]. Another recent study in Ethiopian children revealed only 14.4% and 2.6% of protective anti-HBs antibodies (>10 IU/mL) among under-5-year-olds and in *≥*10-year-old children [14]. There is a rising belief that the presence of breakthrough infections among children previously vaccinated may suggest a genuine lack of response rather than a mere drop in antibody titers below the protective levels [13–19].

Since Uganda integrated the hepatitis B vaccine into UNEPI in 2002, we assumed that persistent detectable anti-HBs antibodies among adolescents (15–17 years) would be a good surrogate for the effectiveness of the immunization program [20], at least in our study jurisdiction. Fledgling emancipation, combined with peer pressures, exposes this age group to behaviors (such as sexual activity, body tattooing, and sharing of injectables) associated with an increased risk of HBV infection [20–23]. HBV seroprevalence in such populations with a heightened risk of infection must be keenly monitored for effective evaluation of vaccination programs to inform adequate control and/or elimination of HBV infection [1,2]. Although acceptable national coverage for every vaccine is 90% [24,25] the Uganda Demographic Health Survey (UDHS-2016) indicated a low (79%) immunization coverage of the childhood hepatitis B vaccine [24]. However, the periodic outbreaks of HBV and the stagnated HBV seroprevalence in Uganda [4,25–27] justify the need to investigate the seroprotective efficacy of the HBV vaccine among children under 5 who have been vaccinated. Further, there is a paucity of data on both qualitative and quantitative determination of HBV serostatus among vaccinated children in Uganda. To the best of our knowledge, immune responses among the children previously vaccinated are variable and inconclusively studied in Uganda [4,26–29]. This study therefore sought to characterize the seroprevalence of HBV vaccine and/or serological status of HBV exposure among children under 5 years and 15–17 years of age. We used qualitative HBV Rapid Diagnostic tests as a baseline for the quantitative estimation to infer the HBV vaccination protection in Kawempe Division, Kampala, Uganda.

## 2. Materials and Methods

### 2.1. Study Design, Population, and Settings

In a cross-sectional study, we enrolled 789 children between May and August 2023 from the Child and Family Foundation (CFU) medical center catchment area in Kawempe Division. The CFU medical center catchment area is located in Kawempe division, one of the five divisions of Kampala city. Kawempe division has 22 administrative parishes. CFU Medical Center conducts both statistics and mobile immunization services under the UNEPI. The statistical immunization services are scheduled for Monday and Friday, while the outreaches are conducted twice a month in the different parishes. All immunization services provided are recorded in the Ministry of Health’s (MOH) health management information system (HMIS). Our study population included children under 5 who had received the hepatitis B vaccine as indicated by the child health card and adolescents (15 to 17 years) who received the hepatitis B vaccine during childhood.

### 2.2. Inclusion and Exclusion Criteria

We included children under five years of age who had received at least one of the three doses of the HBV vaccine, as evidenced by their UNEPI immunization card. For the 15–17-year-old participants, we included participants who had received at least one Hepatitis B vaccine dose verifiable with a UNEPI immunization card or, if not available, based on parents/guardians’ reporting. For both study groups, we excluded children who were anemic as assessed by a study clinician. We also excluded children living with or testing positive for HIV as well as those reporting to have or deemed to have any form of allergy by a study clinician.

### 2.3. Sample Size Estimation and Sampling Procedure

For both group sample sizes, we estimated using Kish Leslie’s formula [30] for cross-sectional studies. We arrived at a sample size of 501 and 288 participants for the under-5 years and 15–17-year-olds, respectively. For the under-5-year-old participant group, we enrolled consecutively eligible children seeking services at the CFU medical center until the sample size was accrued. For the 15–17-year-old study group, eligible study participants were identified during CFU’s scheduled health home visits and community medical outreaches and were referred to the medical center for consenting and conduct of other study procedures until the sample size accrued consecutively.

### 2.4. Data Collection Procedures

A pretested structured questionnaire was administered to parents/legal guardians of participants in the under-5-year-old study group and directly to the participants in the 15–17-year-old group with a provision for consulting their parents/legal guardians if they wished. The questionnaire collected data on the children’s socio-demographic characteristics, such as age, gender, medical history, and hepatitis B vaccination status.

### 2.5. Sample Collection and Processing

Blood was collected from all participants by a designated study clinician or phlebotomist following pre-developed standard operating procedures (SOPs). For the under 5-year-old participants, 8 mL of venous blood was aseptically collected by venipuncture in the following order: 1 EDTA anticoagulant tube of 4 mL, 2 mL Heparinized blood (Becton and Dickson Co. Ltd., Nairobi, Kenya), and a 2 mL plain serum separation tube (Becton and Dickson Co. Ltd., Nairobi, Kenya). For the 15–17-year-olds, 4 mL of venous blood in a plain serum separation tube was collected. Serum and Plasma separation was then conducted by centrifugation of the blood tubes at 1500*× g* for 15 min at room temperature at the Immunology Laboratory of the School of Biomedical Sciences, Makerere University, Kampala, Uganda. All sera, plasma, and PBMCs recoverable were stored in labeled cryovial tubes in volumes of 0.5 mL at –20 °C or lower until analysis at the Integrated Biorepository of H3Africa-Uganda (IBRH3AU), Makerere University, Kampala, Uganda.

### 2.6. Laboratory Analysis

Serum samples were tested for the qualitative detection of HBsAg, HBsAb, HBeAg, HBeAb, and HBcAb using three different Hepatitis B Combo RDT test kits: NOVA TEST-One Step Diagnostic Rapid Test (Global Science, Malaysia; Jaya, Selangor D.E., Malaysia, Cat#: 20221811HBV), Fastep Rapid Diagnostic Test (Healgen Scientific Limited Liability Company, Houston, TX, USA, Cat#: 21076412), and Beright Diagnostic Rapid Test (Medhealth Biotechnology Limited, Shanghai, China, Cat#: 12216453), with manufacturer reported sensitivity and specificity of (91.43% and 98.28%), (68% and 77%), and (98.92% and 99.46%), respectively. Positive and negative controls (prepared and distributed to health facilities by the Uganda Virus Research Institute (UVRI)) were included in all kits and testing runs. Children who tested positive for HBsAg were referred to the CFU Medical Center for appropriate clinical management.

### 2.7. Data Analysis

Interpretation of HBV infection status was indicated in Supplementary Table S1 and as described in Section 2.8 below. All the participants’ data for hepatitis B Combo RDT results were consistent on all three test kits and were therefore included in the analysis. Data were entered for cleaning into EpiData software version 3.1 and exported to SPSS version 25 for descriptive analysis. The results were presented as frequency tables and graphs. The main outcome of interest was HBV serostatus, which was expressed as a percentage. Concordance among the 3 serological tests was analyzed according to Langton et al. (2002) as the percentage of chance that they would yield the same results [31] (Supplementary Table S2).

### 2.8. Interpretation of the Hepatitis B Serological Test Results

Interpretation of serological results in relation to HBV exposure or vaccine status was performed according to the Australasian Society for HIV, Viral Hepatitis, and Sexual Health Medicine (ASHM)’s 2024 Clinical Tool for Decision Making in Hepatitis B (www.testingportal.ashm.org.au/hbv, accessed on 24 May 2024). Study participants with detectable HBsAg were considered HBV infected, with additional positive anti-HBc or HBeAg detected interpreted as having chronic or acute HBV infection, respectively. Participants who were negative for HBsAg, anti-HBc, with or without detectable anti-HBs were considered to have never been exposed to HBV infection. Those participants who were positive for anti-HBc and were also either positive or negative for both HBsAg and anti-HBs were considered to have ever been exposed to HBV infection. However, participants who were negative for HBsAg and positive for anti-HBc and positive or negative for anti-HBs were considered to have ever been exposed to and resolved HBV infection (Supplementary Table S1).

## 3. Results

### 3.1. Socio-Demographic Characteristics

A total of 789 children participated in this study: 288 and 501 in the 15–17-year-old and under 5-year-old groups, respectively. The mean age of this study participants was 16.18 years and 2.93 years for the 15–17-year-old and 0–5-year-old groups, respectively. While more female children (51.7%) were enrolled in the under-5-year-old group, more males (51.74%) were enrolled in the 15–17-year-old group.

### 3.2. Concordance Among NOVA TEST, Fastep, and Beright Diagnostic Rapid Tests

Testing all this study participants blood sera (*n* = 789) with each of the three hepatitis B Combo RDT tests yielded 100% concordance and a similar number of positive and negative results for the five HBV seromarkers: HBsAg (12), anti-HBs (219), HBeAg (7), anti-HBe (5), and HBcAb (6) (Supplementary Table S2).

### 3.3. Doses of Hepatitis B Vaccine Received

Of the 789 study participants, 760 (96.32%) received a verified and documented number of the HBV vaccine doses; 100% (501 of 501) and 89.93% (259 of 288) among the 0–5 and 15–17-year-old groups, respectively. Of the verified vaccine recipients (*n* = 760), 629 (82.76%), 79 (10.39%), and 52 (6.84%) received 3, 2, and 1 dose of the HBV vaccine, respectively.

Notably, relatively more children in the 0–5-year-old group (88.02% [441 of 501]) than those in the 15–17-year-old group (72.59% [188 of 259]) received the 3 HBV vaccine doses, unlike the 1 and 2-dose vaccine recipients who were predominant in the 15–17-year-old group (Table 1).

### 3.4. Qualitative Anti-HBs Status and Prevalence of HBV Infection Among Study Groups

**Figure 1.**
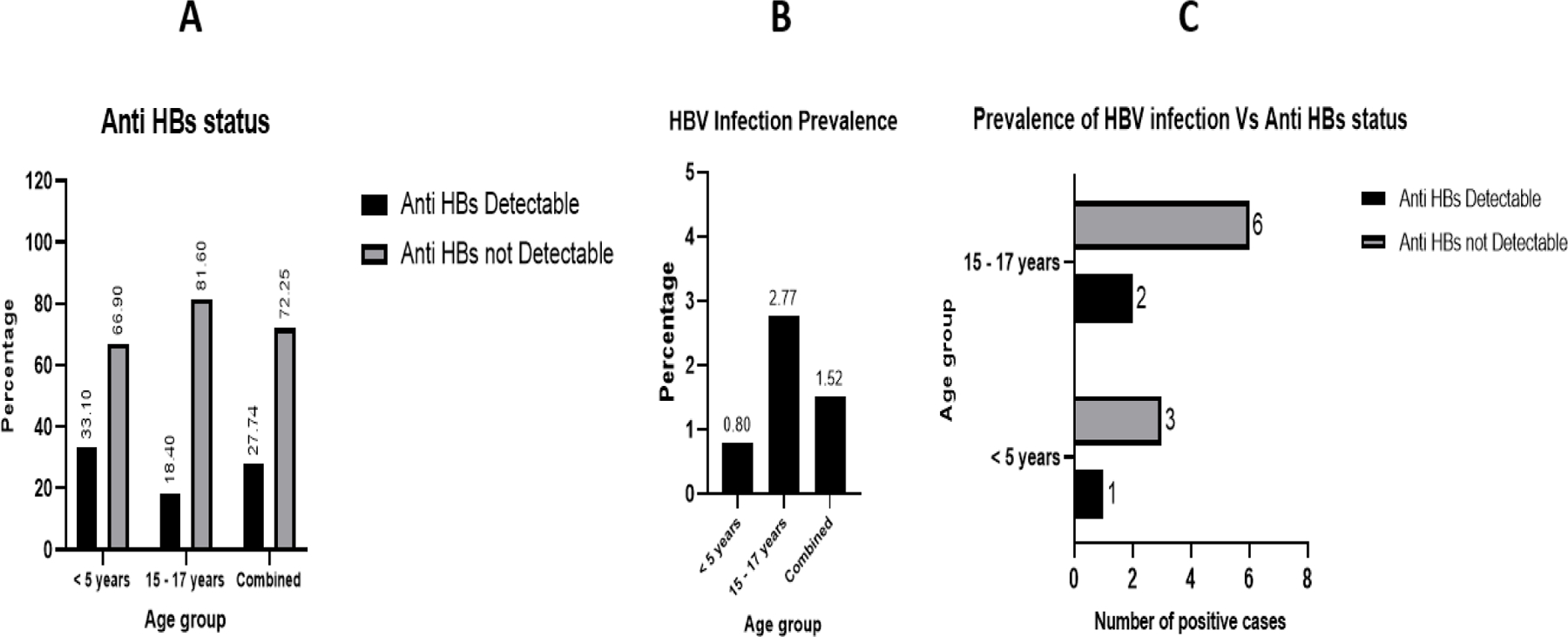
Hepatitis B virus (HBV); (**A**) surface antibody (anti-HBs); (**B**) surface antigen (HBsAg); (**C**) HBsAg and anti-HBs; serostatus, among the study participants Kawempe Division, May-August 2023.

Among the entire study participants, 27.75% (219 of 789) had detectable anti-HBs; 33.10% of the children in the 0–5-year-old age cohort (166 of 501) and 18.40% (53 of 288) in the 15–17-year-old age cohort, respectively (Figure 1A). The overall prevalence of HBsAg (active HBV infection) among all study participants was 1.52% (12 of 789); 2.77% (8 of 288) and 0.80% (4 of 501) in the 15–17-year-old and 0–5-year-old groups, respectively (Figure 1B). Acute HBV infection was demonstrated in 25% (2 of 8); the rest (6 of 8) of the 15–17-year-old participants were chronically HBV infected. All 100% (4 of 4) of children 0–5- years -old were acutely HBV infected; that is, none had chronic infection at the time of study (Table 2). There was a significant difference in proportion of HBV infection between the 15–17 and 0–5 year-old age groups (*p* = 0.087).

Two of the 8 (25%) children infected with HBV (qualitatively detectable HBsAg) among the 15–17-year-old group also had qualitatively detectable anti-HBs on the HBV Combo cassette. Also, in the 0–5-year-old group, one (25%) out of the 4 children with HBV infection had qualitatively detectable anti-HBs on the HBV Combo RDT cassette (Figure 1C). In the entire study population, 72.24% (570 of 789) did not possess detectable anti-HBs; 68.86% (335 of 501) in the 0–5-year-old and 81.59% (235 of 288) in the 15–17-year-old groups, respectively (Table 2).

Of the 11 children in the 15–17-year-old group who had evidence of ever being exposed to HBV, 27.3% (3 of 11) had serological evidence of having resolved the infection. However, none (0 of 4) of the children in the 0–5-year-old group who had ever been infected by HBV had serological evidence of having resolved the infection. Overall, 98.09% (774 of 789) of children in the entire study population had not been exposed to HBV infection before; 99.2% (497 of 501) and 96.18% (277 of 288) in the 0–5 and 15–17-year-old groups, respectively (Table 2).

## 4. Discussion

This study aimed to determine the proportion of vaccinated children with qualitatively detectable anti-HBs antibodies in Kampala, Uganda. In 2016, the World Health Assembly (WHA) adopted a resolution for the elimination of viral hepatitis by 2030, setting elimination targets of a 90% reduction in incidence and a 65% reduction in mortality for hepatitis B by 2030 [32]. Expanding both vaccine coverage and third dose completion to more than 90% of infants, diagnosing more than 90% of estimated chronic hepatitis virus infections, and enrolling them in care are key national-level strategies for the elimination of HBV infections.

Uganda integrated the HBV vaccine into UNEPI in 2002 [26,33], continuously increasing coverage over time. Our teenage and under 5-year-old study participants (average age 16.2 and 2.9 years, respectively) received the hepatitis B vaccine in 2006–2008 and 2018–2020, respectively, approximately 10–12 years apart. In this timeframe, our results suggest a 16% increase in hepatitis B vaccine third dose coverage (from 72.6% to 88%), with an approximate halving of the numbers of children stopping at the first and second dose(s). Our data compares with the WHO/UNICEF Estimates of National Immunization Coverage (WUENI) of 2008 and 2020 (71% and 89% for the 3rd dose in 2008 and 2020, respectively) [34,35], affirming Uganda’s commitment to improving hepatitis B vaccine coverage to meet the global targets. Although the 2020 third dose coverage fell below the 90% global target, official data shows that Uganda surpassed and consistently maintained third dose coverage at 93% and above [36] by 2016. The 2020 drop in coverage coincides with the COVID-19 pandemic, which disrupted healthcare systems globally, including vaccination services [37].

The Hepatitis B vaccine is known to be very efficacious, and response rates are high, ranging between 85% and 100%, eliciting antibody titers far and above the 10 mIU/mL protective limit [38]. However, asymptomatic breakthrough infections (detected by the presence of anti-HBc, anti-HBs, or HBV DNA in serum) have been reported in vaccinated persons with a documented initial antibody response [39,40]. Age at infection greatly determines whether HBV infection progresses into a chronic state: about 90% of infants will develop chronic infection when infected within the first year of life, compared with 30% of children aged 1–4 years and less than 5% of adults [41]. Therefore, it is of paramount importance that vaccine responses in infancy can prevent HBV infection. To meet the global targets for the elimination of HBV infections, ideally, countries would need to perform anti-HBs titer determination to confirm elicitation of protective levels among vaccine recipients, as is the case in vaccinated workers at risk [42]. The anti-HBs titer test availability as well as cost are prohibitive in low- and middle-income countries (LMICs) and would translate into huge programmatic costs, a situation that may divert funds meant for expanding and maintaining optimal vaccination coverage. On the contrary, qualitative strip or cassette-based chromatographic tests that detect anti-HBs antibodies (singly or in combination with other markers of HBV infection) are readily and cheaply available in LMICs. While qualitative anti-HBs cassette tests may not give levels of anti-HBs titers, in resource-limited settings they may be a surrogate for the tittering methods. Using this assumption and qualitative testing, we were surprised to find that only one-third (33.1%) of vaccinated children under 5 years and 18.4% of the 15–17-year-old participants had qualitatively detectable anti-HBs antibodies. Both proportions seemed to be very low for such populations at high risk for HBV infection [2], but are in accordance with findings elsewhere. Long-term follow-up studies of persons vaccinated as infants have reported the absence of detectable anti-HBs antibodies in 50–70% of persons 15–30 years later [39,43–45]. In the African setting, a recent study by Alemayehu, T et al., 2024, found that only 14.4% of children had protective anti-HBs antibodies [14]. Equally, our systematic review of 2023 reported low seroprotection rates across Africa [20]. Taken together, these results confirm low proportions of detectable anti-HBs antibodies in our study groups, which should concern Uganda’s hepatitis B vaccination program as studies have reported a lack of anamnestic responses in close to half of those vaccinated in infancy [43], and infections continue to be reported in children in as short a period as 5 years post completion of vaccination [46]. Contrary to our findings, some studies have reported moderate to high seroprotection rates for children under five years of age [47–53] and among teenagers [21–23,54–57] respectively. Several factors could have contributed to this notably improved vaccine efficacy, including administration of birth doses and enhanced vaccine coverage in this study setting.

Our study also showed HBV infection prevalence of 0.8% among the under-5-year-olds, which more than tripled to 2.77% in the 15–17-year-old participants. While all four infected under-5-year-olds were still acutely infected, six of the eight HBV-infected 15–17-year-olds were chronically infected, demonstrating the high tendency of pediatric HBV infections to progress into a chronic state [41]. A higher prevalence in adolescents versus those under 5 years is in line with the chronicity of pediatric infections and the evidence that adolescents are more likely to have low seroprotection [14], hence more prone to breakthrough infections. Adolescence-associated socio-behaviors are also known to increase the risk of HBV exposure and infection [58–60]. Our study’s 2.77% prevalence in adolescents is comparable to those reported in studies among similar age groups that mostly range between 2.3–2.6% [13,14,61,62]. However, the seroprevalence of HBV infection reported by our study is relatively higher than that reported in low (Ethiopia; 0.4% [63], Egypt; 0.6% [64], Egypt; 0.4% [65]) and high-income countries (Italy; 0.6% [66], Iran; 0.6% [67], Uzbekistan-Japan; 0.8% [68]). The varying levels of breakthrough infections may be attributed to EPI vaccination coverage rates and evaluation, host immunogenetic factors, vaccination schedule adopted, and the method used to detect HBsAg.

A combined total of fifteen breakthrough infections is testament to suboptimal levels of protection induced by the hepatitis B vaccine in some members of both groups or of waning levels of protection [39,43]. In Uganda, 5–17% of vaccine recipients are known to be non-responders regardless of completion of all 3 doses [28,69]. Our data showed that the majority of those with active HBV infection (6 of 8, and 3 of 4 among 15–17 year and under 5-year groups, respectively) were among those with no qualitatively detectable anti-HBs antibodies, pointing to the fact that suboptimal or non-response to, or significant waning of vaccine responses leaves children susceptible to HBV infection. This is supported by Reda AA et al.’s (2003) study in Egyptian 6-year-old children [70] that reported a three-fold higher prevalence of HBsAg positivity (2.2% versus 0.8%) among vaccine non-responders in comparison to those with detectable anti-HBs.

Results of this study strengthen our idea that in the absence of access to tittering methods or financial constraints, qualitative Rapid Diagnostic Tests-based methods for the detection of anti-HBs antibodies may be valuable in identifying those susceptible and at high risk of HBV infection. The extent of utility of such qualitative anti-HBs detection methods may be better among children who are recent recipients of the vaccine and therefore expected to still have high levels of vaccine-induced anti-HBs antibodies detectable even on the less sensitive cassette-based RDT methods. Determination of the true utility of these qualitative RDT cassette-based methods would require studies to determine lower limits of detection [71,72], based on anti-HBs antibody titers. Clinical trials that estimate the proportion of vaccinated children with titers below detection levels who would otherwise be missed by relying on the qualitative method alone would also provide the public health risk of such an approach. Such studies would provide the extent of measurement uncertainty or confidence with which to credit qualitative test results concerning screening vaccine immune responses or susceptibility to HBV infection.

Rapid Diagnostic Test-based technologies (RDTs) have dominated Africa’s clinical laboratories and expanded access to cheap, timely, and reliable diagnostic services in the region, including at point-of-care settings. The challenge of RDT-based tests is the lack of national-level policy or validation data to guide the choice of a better test brand for use, market stockouts of validated test kit brands, and the rapid emergence of new brands altogether. Laboratories implementing quality management systems do carry out some level of performance verification of emerging RDT brands at a local level, but the high turnover of brands on the market makes the exercise daunting. We provide reassuring data that the three brands used in this study were concordant, and therefore may be used interchangeably to address stockouts in the Ugandan market.

## 5. Conclusions

We report a low prevalence of children with qualitatively detectable anti-HBs antibodies (33.3% among under-5-year-olds and 18.4% among 15–17-year-olds). The proportions above and being much lower in the 15–17-year-old participants are evidence of a rapid waning of anti-HBs antibodies over time. The majority of HBV infections are happening in those with no detectable anti-HBs antibodies, which affirms increased susceptibility to infection as well as a tendency to progress into the chronic state in children. The three Hepatitis B Combo test kit brands (NOVA TEST, Fastep, and Beright Diagnostic Rapid Tests) were perfectly concordant for all markers of HBV infection. Anti-HBs titer determination and the determination of the extent of the utility of qualitative anti-HBs methods were outside the scope of this study but are recommended in the future or for other researchers to undertake.

## 6. Limitations

Whereas our study boasts a good sample size, this study was conducted in only one of the five administrative divisions of Kampala and may not represent the entire city or be extrapolable to the rest of Uganda. Additionally, we were unable to perform quantitative measurements of anti-HBs titers. We also did not conduct HBV viral load testing; instead, participants found to be infected were referred to a public health facility for specialist care, usually provided at no cost to the patient. Also, not all of this study participants enrolled in our study completed all three WHO-recommended HBV vaccine doses. Finally, we did not determine and exclude congenital exposure to HIV, a factor that is known to affect quality and magnitude of immune responses in a subsection of exposed infants.

## Supplementary Material

Supp 1

Supp 2

The following supporting information can be downloaded at: https://www.mdpi.com/article/10.3390/livers4040039/s1. Table S1: Interpretation of the hepatitis B serological test results; Table S2: Concordance among the HBV Rapid Diagnostic Tests.

## Figures and Tables

**Table 1.** Characteristics of this study participants, Kawempe Division, May-August 2023.

Variables	15–17 Years, *n* = 288	0–5 Years, *n* = 501	Combined, *n* = 789

**Age Mean (SD)**	16.18 (0.8836)	2.93 (1.3)	
**Gender *n* (%)**			
Male	149 (51.74)	242 (48.3)	391 (49.55)
Female	139 (48.26)	259 (51.7)	398 (50.44)
Doses	15–17 years, *n* = 259	0–5 years, *n* = 501	Combined, *n* = 760
One	26 (10.04)	26 (5.19)	52 (6.84)
Two	45 (17.37)	34 (6.79)	79 (10.39)
Three	188 (72.59)	441 (88.02)	629 (82.76)

**Table 2.** Clinical test characteristics of this study participants, Kawempe, May-August 2023.

Variables	15–17 Years, *n* = 288	0–5 Years, *n* = 501	Combined, *n* = 789
**HBV markers combo *n* (%)**			
HBsAg	8 (2.78)	4 (0.8)	12 (1.52)
HBsAb	53 (18.4)	166 (33.1)	219 (27.75)
HBeAg	3 (1.04)	4 (0.8)	7 (0.88)
HBeAb	2 (0.69)	3 (0.6)	5 (0.63)
HBcAb	6 (2.08)	0 (0)	6 (0.76)
**Infection status *n* (%)**			
Never been exposed	277 (96.18)	497 (99.2)	774 (98.09)
Ever been exposed	11 (3.8)	4 (0.80)	15 (1.9)
Exposed and resolved	3 (1.04)	0 (0)	3 (0.38)

## Data Availability

All this study-generated data are included in this research article and the Supplementary Materials.
