# Supplementary material for: Serological Status of Vaccine and Hepatitis B Virus Exposure Among Children Under 5 and Aged 15–17 Years in Kampala, Uganda
Source: Livers. Author manuscript; Available in PMC 2025 Aug 21. (PMC12366772; doi:10.3390/livers4040039)
Supplement: Supp 1 [file NIHMS2091135-supplement-Supp_1.docx]

Serological Status of Vaccine and Hepatitis B Virus Exposure Among Under 5 and in 15-17-Year-old Children in Kampala, Uganda

Author List; Fahad Muwanda, Edward Kiyonga, Joan Nambafu, Hussein Mukasa Kafeero, Edgar Kigozi, Harriet Mupere Babikako, Enock Wekiya, Gerald Mboowa, David Patrick Kateete, Hakim Sendagire, Paul Norman, and Bernard Ssentalo Bagaya

* Correspondence: [bernard.bagaya@mak.ac.ug](mailto:bernard.bagaya@mak.ac.ug)

[muwandafahad@gmail.com](mailto:muwandafahad@gmail.com)

**Supplementary Table 1.** Interpretation of the hepatitis B serological test results.

| **SN** | **HBV seromarkers** | | | | **Serological interpretation** | **Immune status** |
| --- | --- | --- | --- | --- | --- | --- |
|  | **HBsAg** | **Anti-HBc** | **Anti-HBs** | **HBe** |  |  |
| 1. | Positive | Positive | Negative/Positive | Negative/Positive | Chronic HBV infection | Non- immune |
| 2. | Positive | Negative/Positive | Negative/Positive | Positive | Acute HBV infection | Non-immune |
| 3. | Negative | Negative | Negative/Positive | Negative | Never been exposed | Susceptible or non-immune |
| 4. | Negative/Positive | Positive | Negative/Positive | Negative/Positive | Ever been exposed | Immune/non-immune |
| 5. | Negative | Positive | Negative/Positive | Negative | Exposed and resolved | Immune |

[www.testingportal.ashm.org.au/hbv](http://www.testingportal.ashm.org.au/hbv)
