# Supplementary material for: Serological Status of Vaccine and Hepatitis B Virus Exposure Among Children Under 5 and Aged 15–17 Years in Kampala, Uganda
Source: Livers. Author manuscript; Available in PMC 2025 Aug 21. (PMC12366772; doi:10.3390/livers4040039)
Supplement: Supp 2 [file NIHMS2091135-supplement-Supp_2.docx]

Serological Status of Vaccine and Hepatitis B Virus Exposure Among Under 5 and in 15-17-Year-old Children in Kampala, Uganda

Author List; Fahad Muwanda, Edward Kiyonga, Joan Nambafu, Hussein Mukasa Kafeero, Edgar Kigozi, Harriet Mupere Babikako, Enock Wekiya, Gerald Mboowa, David Patrick Kateete, Hakim Sendagire, Paul Norman, and Bernard Ssentalo Bagaya

* Correspondence: [bernard.bagaya@mak.ac.ug](mailto:bernard.bagaya@mak.ac.ug)

[muwandafahad@gmail.com](mailto:muwandafahad@gmail.com)

**Supplementary Table 2.** Concordance among the HBV Rapid Diagnostic Tests.

| **Serial Number** | **Rapid Diagnostic Test** | **HBV markers (n=789): number positive (%)** | | | | |
| --- | --- | --- | --- | --- | --- | --- |
|  |  | HBsAg | Anti-HBs | HBeAg | Anti-HBe | Anti-HBc |
| **1.** | **NOVA TEST** | 12 (1.52) | 219 (27.75) | 7 (0.88) | 5 (0.63) | 6 (0.76) |
| **2.** | **Fastep** | 12 (1.52) | 219 (27.75) | 7 (0.88) | 5 (0.63) | 6 (0.76) |
| **3.** | **Beright** | 12 (1.52) | 219 (27.75) | 7 (0.88) | 5 (0.63) | 6 (0.76) |
